# The role of immunoflorescence in the physiopathology and differential diagnosis of recurrent aphthous stomatitis

**DOI:** 10.1016/S1808-8694(15)30564-4

**Published:** 2015-10-19

**Authors:** Niels Salles Willo Wilhelmsen, Raimar Weber, Ivan Dieb Miziara

**Affiliations:** 1 PhD student in Otorhinolaryngology - University of São Paulo Medical School (HC-FMUSP), Dentist, collaborator at the Stomatology group at the ENT Department HC-FMUSP.; 2 MD; PhD student in ENT - HC-FMUSP, Preceptor of ENT at the HC-FMUSP.; 3 Associate Professor - ENT - HC-FMUSP; Physician in charge of the Stomatology group at the HC-FMUSP.. Grupo de Estomatologia - Ambulatório da Divisão de Clínica ORL - Hospital das Clínicas - Faculdade de Medicina da USP.

**Keywords:** aphthous stomatitis, vesiculobullous skin diseases, fluorescence microscopy, pemphigus vulgaris

## Abstract

Recurrent aphthous stomatitis (RAS) is a disease characterized by the periodic appearance of aphthous lesions on the oral mucosa, of which etiology and physiopathology are not well explained. Recent studies with direct immunofluorescence show controversial results. Some reveal that the basic disorder is associated with humoral immunity, while others point to changes in cellular immunity. Atypical forms of aphthous stomatitis may have its differential diagnosis carried out with vesicobullous diseases, such as pemphigus vulgaris.

**Aim:**

Check the presence of immunocomplexes in the mucosa of patients with aphthous stomatitis and the usefulness of the differential diagnosis method with bullous skin diseases.

**Materials and methods:**

23 patients with aphthous stomatitis were prospectively included in the study. There were all submitted to mucosa biopsy under local anesthesia for the removal of two fragments. One of these was sent to histology and, the other to direct immunofluorescence.

**Results:**

The 23 samples from the histology exam revealed an ulcerated inflammatory process. The samples referred to immunofluorescence resulted negative and only one showed the presence of complement in the basal membrane.

**Conclusion:**

Based on our results, we conclude that the patients with RAS do not show deposits of immunocomplexes in their oral cavity mucosa and immunofluorescence is useful in the differential diagnosis between this disease and bullous skin diseases.

## INTRODUCTION

Recurrent Aphthoid Stomatitis (RAS) is a common disease of the oral cavity, affecting about 20% of the world's population^1^, women are more affected than men, and in most cases it starts around the first decade of life.

**Figure 1:**
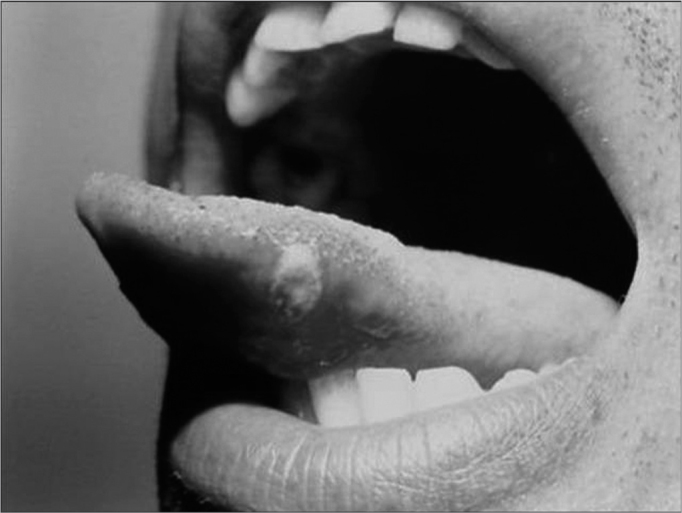
Aphtha minor on the tongue's lateral border

**Figure 2:**
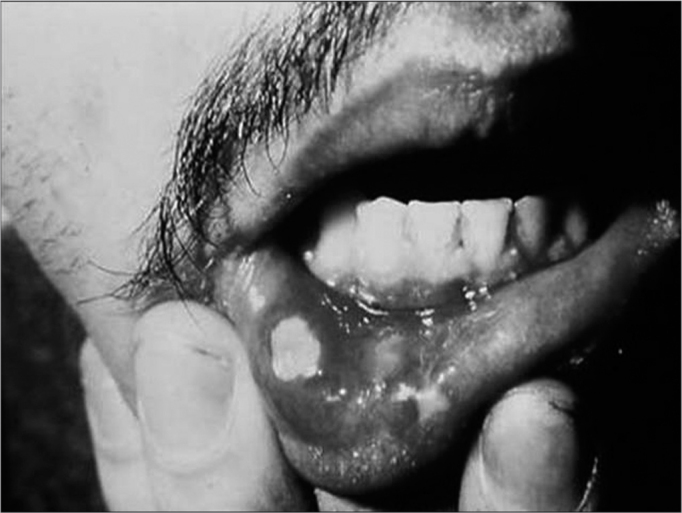
Aphtha major on the lower lip. Notice scar lesions

The disease may manifest itself in three different ways. The minor type is characterized by a small ulcer, measuring from three to ten millimeters in diameter, located in the non-keratinized mucosa, alone or even in large quantities in the mouth^2^, ^3^(Figure 1). The major type is characterized by an extensive and painful ulcer, measuring more than 10 millimeters in diameter, also present in keratinized mucosa, usually alone, and can take more than 15 days to heal completely, and it may leave scars in the mucosa (Figure 2). The third type is herpes-like, characterized by numerous small ulcers that coalesce, and this is the rarest form of the disease^2^, ^3^.

Despite its unknown etiology, many are the factors associated with or that may trigger RAS outbreaks. Among them we list: immune disorders4, blood deficiency^2^, ^3^, ^5^, ^6^, vitamin deficiency^7^, hypersensitivity to food^8^, zinc deficiency^9^, psychological factors^10^, genetic factors^11^, infectious and viral factors^12^, ^13^ and rheumatic disease^14^.

The main physiopathological hypothesis for the origin of RAS lesions are associated with humoral or cellular immunity^4^, ^15^, ^16^, ^17^, ^18^, ^19^, ^20^. Most of the studies aforementioned, among other exams, used direct immunofluorescence (DIf) in order to try to prove the presence or absence of immunocomplex deposits on the mucosa of patients with RAS.

Lehner^15^ and Sistig et al.^4^ advocate the thesis that RAS's physiopathology is associated with a disorder in immunomodulation. These authors did not find positiveness in the direct immunofluorescence test (DIf) in patients with RAS, based on the fact that T-cells are the ones most commonly found in aphthoid lesions. Moreover, another piece of data corroborating this hypothesis is a variation in the ratio of T cells CD4+ / T CD8+ in the oral mucosa affected during its different development stages.

On the other hand, other authors^16^, ^17^, ^18^ found IgG and C3, during DIf, and they advocate that humoral response is present in RAS genesis.

Another important aspect is that, depending on their development stage, aphthoid lesions may be similar to other bullous, auto-immune diseases, such as pemphigus vulgaris (PV) or mucous membrane pemphigoid (MMP)^21^. However, it is known that these diseases are provenly positive in DIf - PV with intra-epithelial deposits of immunoglobulins and MMP with similar deposits in the basal membrane^22^. Thus, DIf in patients with RAS, especially in its typical forms, and if it is shown negative, would have an important role in the differential diagnosis of these diseases.

## OBJECTIVES

1) try to show the presence or absence of immunoglobulins in the perilesional mucosa of patients with RAS, through DIf, in order to add data to the hypothesis that the disease's physiopathology would or would not be connected to the alterations in humoral immunity and
2) check if DIf has a relevant role in the differential diagnosis of RAS and vesicobullous diseases such as PV and MMP.

## MATERIALS AND METHODS

In this present investigation we had 23 consecutive patients with RAS, who reported having at least one episode of aphtha per month, for a minimum period of two years. The study ran from March 2004 to may 2006, with historical and cross-sectional cohort design.

The patients signed an informed consent form and the study was approved by the institution's ethics committee under protocol number 876/04, as part of a larger project to investigate clinical, laboratorial and genetic aspects of patients with RAS.

All patients were submitted to a protocol based on otorhinolaryngological general anamnesis, physical exam, especially that of the oral cavity, CBC, complete coagulogram, serum ferritin level, G6PD (glucose 6-phosphate dehydrogenase) level, anti-nucleus factor (ANF), rheumatoid factor, Lues serology (RSS and Fta-ABS), anti-HIV 1 and 2, serum dosage of immunoglobulins (Ig) A, G and M, C reactive protein.

### Inclusion criteria

- Clinical history matching signs of RAS, i.e, outbreaks of oral cavity aphthoid lesions, with monthly periodicity (or less), for at least two years;
- Aphthoid lesion on the oral mucosa;
- No changes in the exams ordered according to protocol;
- Absence of clinical and/or laboratorial signs matching those of systemic disease with oral lesions.
- No use of topical steroid for at least two weeks before the biopsy.

### Exclusion criteria

- Clinical history different from that of RAS;
- Presence of alterations in the tests ordered in the protocol;
- Clinical and/or laboratorial signs matching those from a systemic disease with oral lesion.
- Use of topical steroid for at least two weeks prior to biopsy.

In order to rule out other diseases that affect the oral cavity and make the differential diagnosis with RAS (Behçet's disease, Pemphigus and Pemphigoid, Erythema multiforme etc.) the lesion was biopsied, a fragment of perilesional tissue was collected for direct immunofluorescence (DIf).

Biopsy was carried out under injected anesthesia with 2% xylocaine without vasoconstrictor, by using a 4mm punch tool, on the mucosa adjacent to the lesion (perilesional) for DIf and another fragment was harvested at the transition area between the ulceration and the lesion area of the mucosa for histology purposes.

The fragment for histology was placed in a proper container with 10% formaldehyde.

The external surface of the perilesional epithelium harvested for DIf, dyed with methylene blue for better identification. The fragment collected was sent in a gauze wet in 0.9% saline solution, to the Skin Immunopathology Laboratory of the Dermatology Department at the HC-FMUSP.

These fragments were frozen with inclusion medium (Tissue Tek, Leica, Germany), wrapped around aluminum foil and taken to the freezer at -20°C until the time for processing.

They were then cut in cryostat, at a temperature of -20ºC (cryo-section). Three cross-sections from each patient, 4 microns-thick were placed on albumin-loaded slides.

For the immune reaction, the slides were placed in a wet chamber, at room temperature, and on the cross-sections we added the conjugated (anti human immunoglobulins produced in immunized animals and marked with fluorescein isothiocyanate). The conjugated material was diluted in TBS-calcium (TBS-Ca++) (“trisma base solution”, pH 7,5) with 3 mg% Evans Blue Dye (Interlab). We used anti human IgA from SIGMA (1:20 dilution); anti-human IgM, also from SIGMA (1:20 dilution); anti human IgG from SIGMA (1:130 dilution) and anti human C3c from DAKO (1:40 dilution). After a 30-minute incubation period, the slides were washed in TBS for 2 ten-minute periods each.

In order to setup the slides we used buffered glycerin (pH 9/ 0.5M) and glass lamina. The slide was read in a epiluminescence HBO 50w Zeiss microscope (CB12 filter), with a 10x eyepiece and 16x and 40x lenses.

## RESULTS

**Figure 3:**
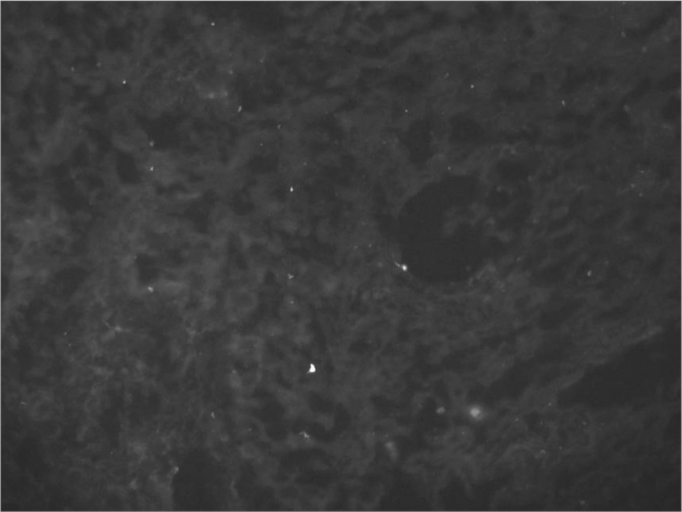
Negative direct immunofluorescence in recurrent aphthoid stomatitis

In our series, nine patients were males and 14 were females. Their ages ranged between 18 and 70 years (mean of 36.14 years, standard deviation of 15.33 years). All histology exams showed the presence of an ulcerated lesion on the mucosa, followed by unspecific chronic and acute inflammatory process on the submucosa. Routine dyeing and the investigation for fungi and BAAR were all negative, as were the immunohistochemical analysis. Of the 23 samples sent for Dif, only one sample showed mild C3 granulous presence in the basal membrane zone. As for IgG, IgM and IgA all the 23 samples did not show specific fluorescence in the basal membrane and the vessel walls (Figure 3).

## DISCUSSION

The major role of direct immunofluorescence is to identify antibodies and other inflammatory proteins in the region damaged by disease and thus confirm or rule out the clinical/diagnostic suspicion of a lesion of etiology associated with humoral immunity disorders^23^.

**Figure 4:**
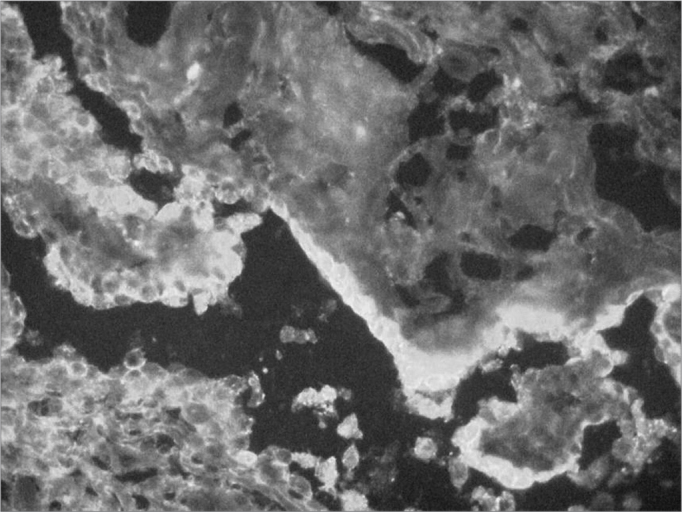
Positive direct immunofluorescence in Pemphigus vulgaris

Therefore, we may suppose that a disease with positive fluorescence for immunoglobulins on the mucosa is associated to disorders in the production of antibodies and it is auto-immune, as it happens with patients with pemphigus vulgaris (Figure 4)^23^.

Therefore, DIf is an excellent test for the differential diagnosis between autoimmune disorders and those that have no relation with changes in humoral immunity^23^. On the other hand, it is worth remembering that a negative fluorescence is not pathognomonic of a disease associated with cell immunity disorders^23^.

In the present investigation we carried out histology tests and DIf in 23 patients with RAS. Our results may be directly compared to those from other authors who used DIF in patients with RAS - as depicted on Table 1. It is important to notice that among all the papers mentioned, ours is the one with the most studies with DIf.

**Table 1:** DIf findings in RAS patients according to data surveyed in the medical literature.

Direct immunofluorescence studies in patients with RAS								
	Vessel walls				Basal membrane zone			
	IgG	IgM	IgA	C3**	IgG	IgM	IgA	C3
Donatsky e Dabelsteen	0/16*	0/16*	0/16*		14/16*	0/16*	0/16*	16/16*
Ullman e Gorlin	0/10*	6/10*	2/10*	10/10*	0/10*	0/10*	0/10*	5/10*
Van Hale et al.	0/22*	1/22*	0/22*	7/22*	1/22*	1/22*	0/22*	6/22*
Malmström et al.	0/16*	0/16*	0/16*	15/16*	-	-	-	-
Reimer et al.	0/17*	4/17*	0/17*	13/17*	0/17*	0/17*	0/17*	6/17*

Wilhelmsen et al.	0/23*	0/23*	0/23*	0/23*	0/23*	0/23*	0/23*	1/23*

* Number of positive cases / number of patients studied

** C3 = complement fraction 3

Donatsky and Dabelsteen^16^ used direct immunofluorescence to study the biopsy of 16 patients with RAS, and they found IgG in the basal membrane of 14 patients, all had C3 deposits and there was no specific fluorescence for IgM and IgA. On the vessel walls there were no IgG, IgM and IgA markers.

Significant presence of C3 in the basal membrane was also found by Ullman and Gorlin^17^. In their study, five of 10 patients had this result; however in this region they did not find fluorescence for IgG, IgM and IgA. Vessel walls were negative for IgG; positive in 6 patients for IgM; in 2 patients for IgA; and all 10 patients were positive for C3.

Studying a population similar to ours, comprising 22 patients, VanHale et al.^18^ did not find IgA on vessel walls, nor in the basal membrane zone. IgG was also negative on vessel walls. IgM was positive in only one case both in the basal membrane as well as on vessel walls. As to the presence of C3, it was positive in six cases on vessel walls and seven on the basal membrane zone.

Malmström et al.^19^ found no fluorescence for IgM, IgG and IgA in all 16 cases assessed; however, in 15 of them there was C3 fluorescence along vessel walls.

In 17 cases, Reimer et al.20 did not find fluorescence on the basal membrane for IgG, IgA and IgM; however, in six cases there was fluorescence for C3. The vessel walls did not present fluorescence for igG and IgA; and fluorescence was present in 4 cases for IgM and 13 cases for C3.

Our study with direct immunofluorescence in 23 patients is in conflict with the findings of these two authors^16^, ^17^ and in partial agreement with those from VanHale et al., Malmström et al. and Reimer et al.^18^, ^19^, ^20^. In our series we could not find specific fluorescence for igG, IgA and Igm on vessel walls and also on the basal membrane zone. In only one case we observed C3 fluorescence, even then it was mild in the basal membrane.

It is important to stress that the patients who participated in this study did not use any drug before the biopsy was made since false-negative results may be found if the region is treated with topical steroids prior to the biopsy^23^.

On the other hand, our results are in agreement with those from Lehner15 and Sistig el al.^4^, who were also unable to prove DIf positiveness in patients with RAS. These authors do not agree with the statement that RAS is a humoral response, they believe it is an ulcerative process triggered by cellular immunity.

As recently described by Natah et al.^24^ Scully et al.^25^, RAS immunopathogenesis involves an immune response mechanism mediated by cells, generation of T-cells and tumor necrosis factor alpha (TNF-α) by other leucocytes (macrophages and mast cells). It is also known that in the ulcerative phase of the disease there is a predominance of CD8+ T cells and a reduction of CD4+ T cells on the mucosa affected.

Aside from this, our findings show that DIf is paramount for the differential diagnosis between the atypical RAS and vesicobullous diseases, which provenly have immunocomplex deposits on the epithelium. As shown by Femiano et al.^21^, there are forms of RAS, especially the major type, that causes large areas of mucosal ulceration and may be easily mistaken with PV and MMP, causing important diagnostic problems, preventing proper treatment in due time

## CONCLUSION

Based on our findings, we conclude that:

1) DIf is negative on the perilesional mucosa of RAS patients. This data strengthens the hypothesis that the disease is especially associated with cell immunity disorders;
2) DIf is important in the differential diagnosis of atypical RAS (when the test is negative) and vesicobullous diseases (when the fluorescence is positive on the oral epithelium).
